# Data for bottom ash and marble powder utilization as an alternative binder for sustainable concrete construction

**DOI:** 10.1016/j.dib.2020.105160

**Published:** 2020-01-23

**Authors:** Mohamad Atiyeh, Ertug Aydin

**Affiliations:** Department of Civil Engineering, European University of Lefke, Lefke, TR-10, Northern Cyprus Turkey

**Keywords:** Marble powder, Cement, Paste, Composite, Sustainability, Construction, Waste

## Abstract

In today's world where the effects of global warming are intense, alternative approaches for a sustainable concrete sector are rapidly increasing. The partial replacement of waste materials, such as bottom ash and marble powder to cement, leads to a significant reduction in carbon dioxide emissions. The expected quality of materials to be used for the concrete sector is very important in terms of building safety. New building materials and binder materials offered as an alternative are often unable to meet this requirement due to existing standards. Therefore, the existing standards should be reviewed and alternative construction materials should be provided for the sector. This dataset would be beneficial for other researchers who design sustainable concrete. Although the data presented herein deals with pure paste, the authors believe that the data can be used for mix design in the concrete construction sector to reduce the cement amount for various civil engineering projects, from low to medium strength concrete applications. The data compiled herein were obtained from the fresh and hardened properties at 7, 28 and 56 days. Physico-mechanical characterization and sulphate resistance were analyzed for laboratory produced marble paste composites. The dataset described here is the pilot study of the “High-volume marble substitution in cement-paste: towards a better sustainability” [1] and “Novel coal bottom ash waste composites for sustainable construction” [2].

**Table:** Specifications Table

Subject	Materials Science (General), Engineering
Specific subject area	Civil and Structural Engineering
Type of data	Table, Image, figure, video, text file
How data were acquired	Physical, mechanical and sulphate tests at the age of 7, 28 and 56-days of hardening. Nonlinear regression analysis and data plotting software were used to evaluate the laboratory results.
Data format	Raw, analyzed
Parameters for data collection	Four different mixture groups composed of marble powder (20 and 25% by weight) and bottom ash (20 and 25% by weight) were used. The water to binder ratio was kept constant at 36.5%. The composites were tested at 7, 28 and 56-days of hardening.
Description of data collection	Data was obtained from laboratory experiments at the age of 7, 28 and 56-days of hardening by mini slump, flow table, water absorption, apparent specific gravity, bulk specific gravity, porosity, unconfined compressive strength, flexural strength and sodium sulphate tests.
Data source location	TR- 10 Turkey, Lefke, Northern Cyprus
Data accessibility	The data presented herein and supplementary data files are available within this article.
Related research article	Aydin, E; Hasan A.Ş, High-volume marble substitution in cement-paste: towards a better sustainability, J. Cleaner Prod. 2019, 237C, 10.1016/j.jclepro.2019.117801 Aydin E. 2016. Novel Coal Bottom Ash Waste Composites for Sustainable Construction, Constr. Build. Mater.124, 582–588.

**Table:** 

**Value of the Data** • This data is composed of alternative material for building construction. • The researcher can combine the current data with the other data described in Ref. [3] to produce a wide range of paste groups composed of marble powder. • The bottom ash can also be used for the same methodology for better sustainable construction as an alternative binder material for concrete production. • This data provides significant carbon dioxide reduction in civil engineering applications.

## 1 Data

**Fig. 1:**
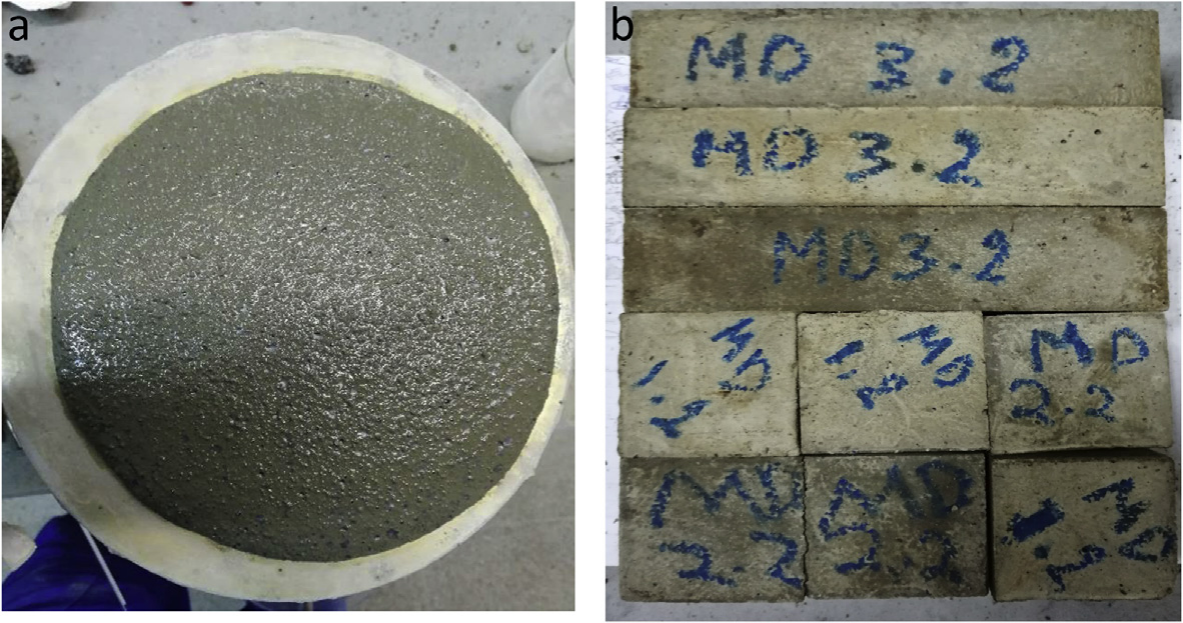
**a.** Flow table test for workability. **b.** Selected samples.

**Fig. 2:**
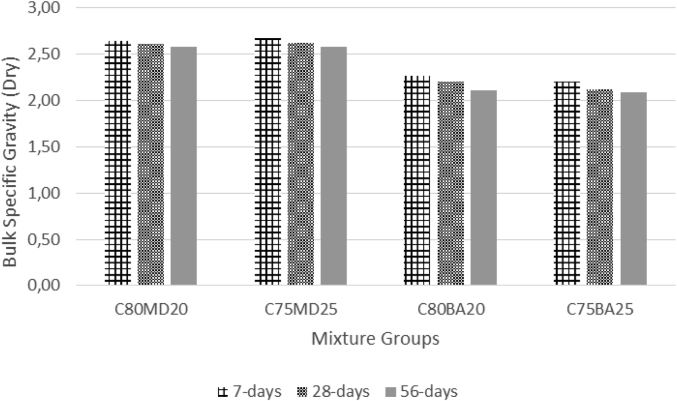
Bulk specific gravity (dry) for bottom ash-marble-cement paste composites at 7-28-56 days of hardening.

**Fig. 3:**
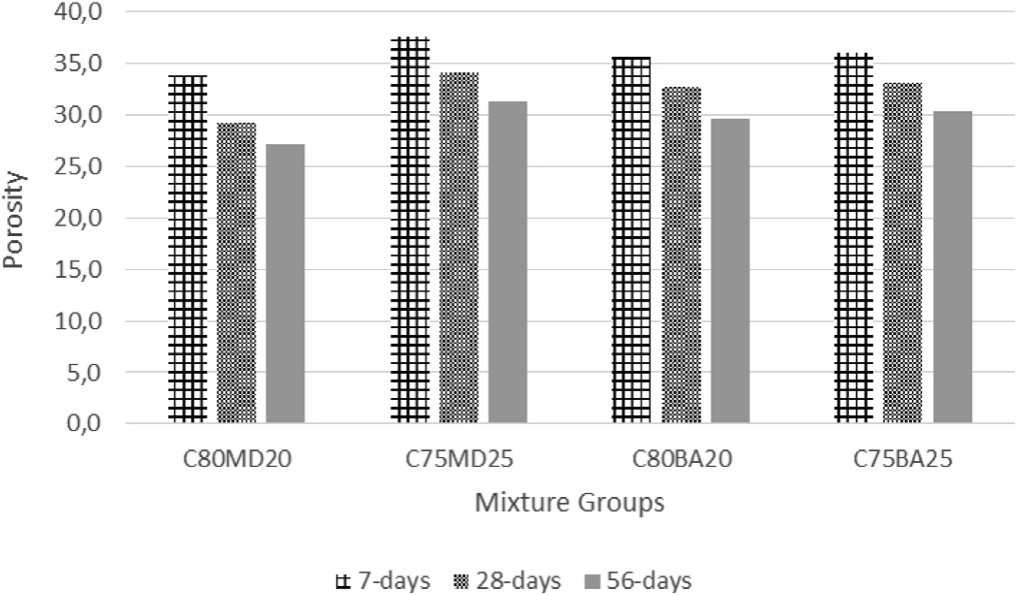
Porosity values for bottom ash-marble-cement paste composites at 7-28-56 days of hardening.

**Fig. 4:**
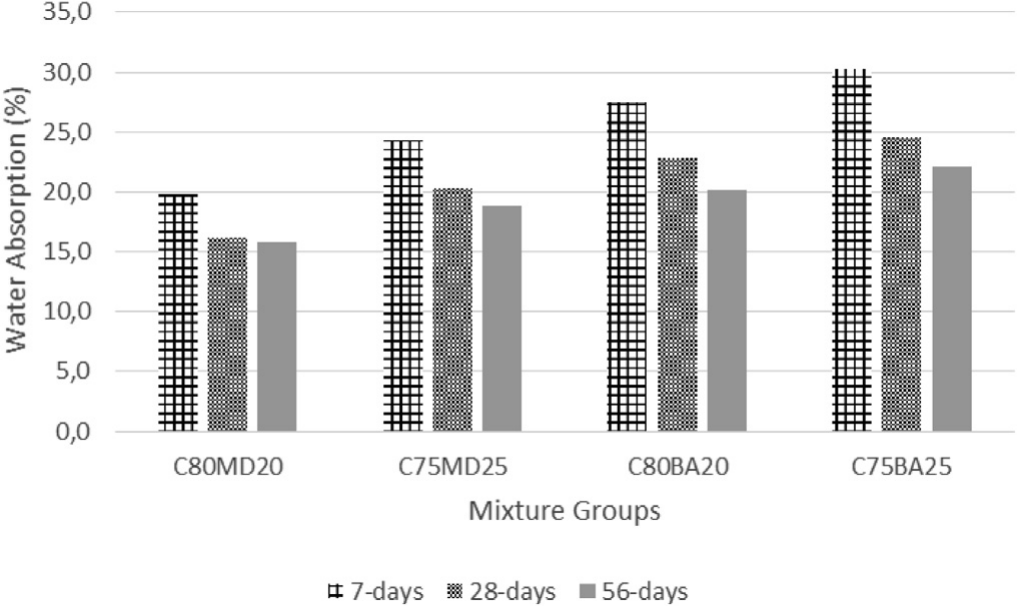
Water Absorption values for bottom ash-marble-cement paste composites at 7-28-56 days of hardening.

**Fig. 5:**
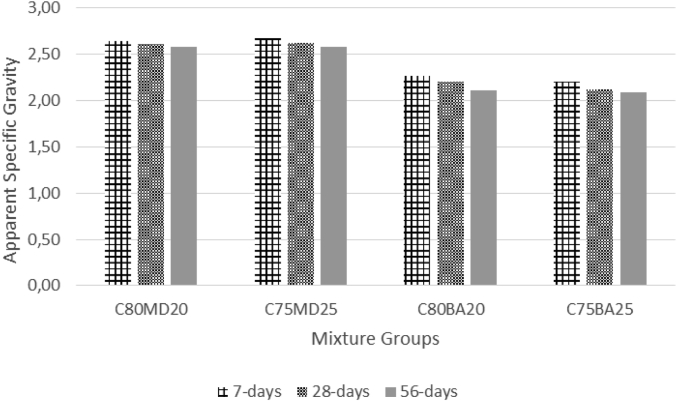
Apparent specific gravity for bottom ash-marble-cement paste composites at 7-28-56 days of hardening.

**Fig. 6:**
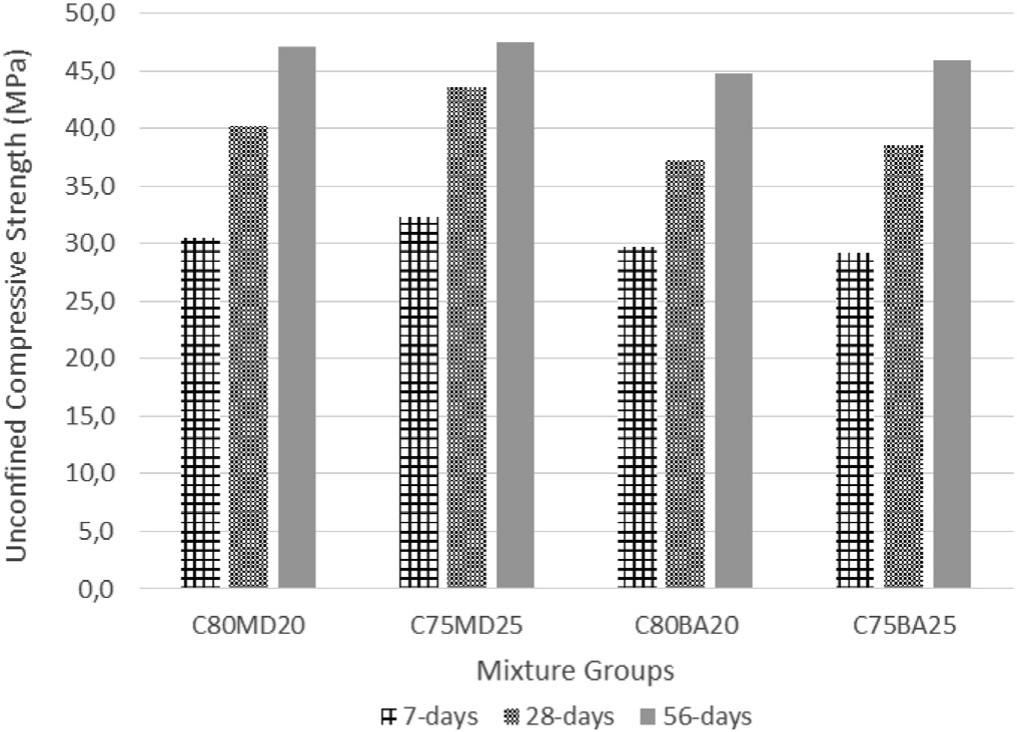
Compressive strength values for bottom ash-marble-cement paste composites at 7-28-56 days of hardening.

**Fig. 7:**
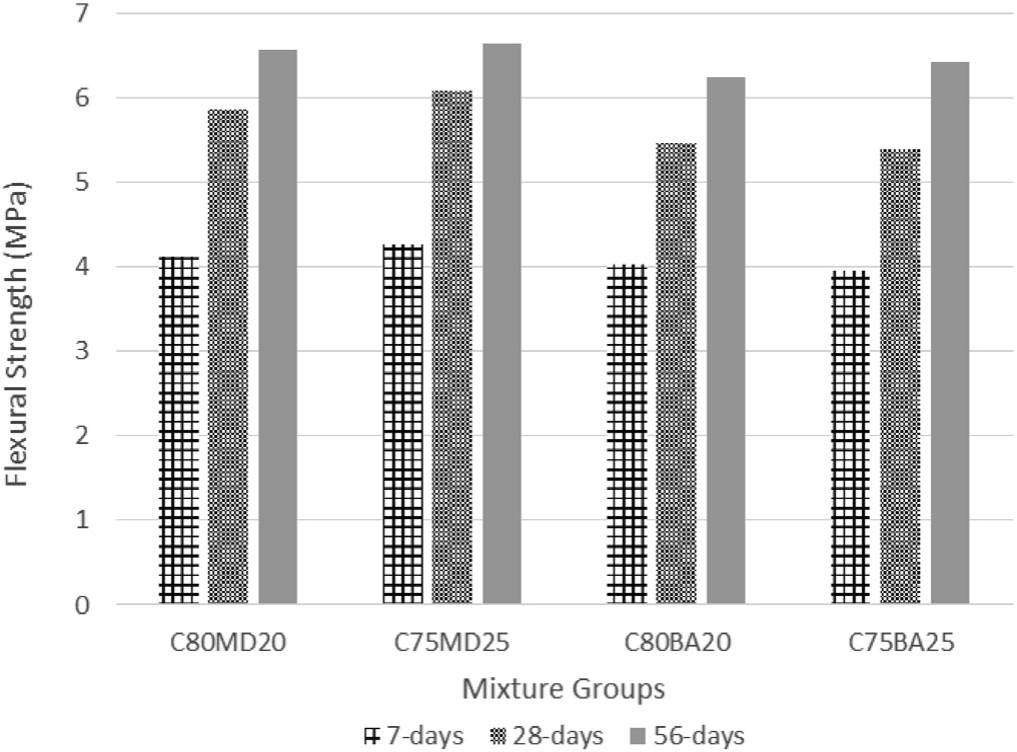
Flexural strength values for bottom ash-marble-cement paste composites at 7-28-56 days of hardening.

**Fig. 8:**
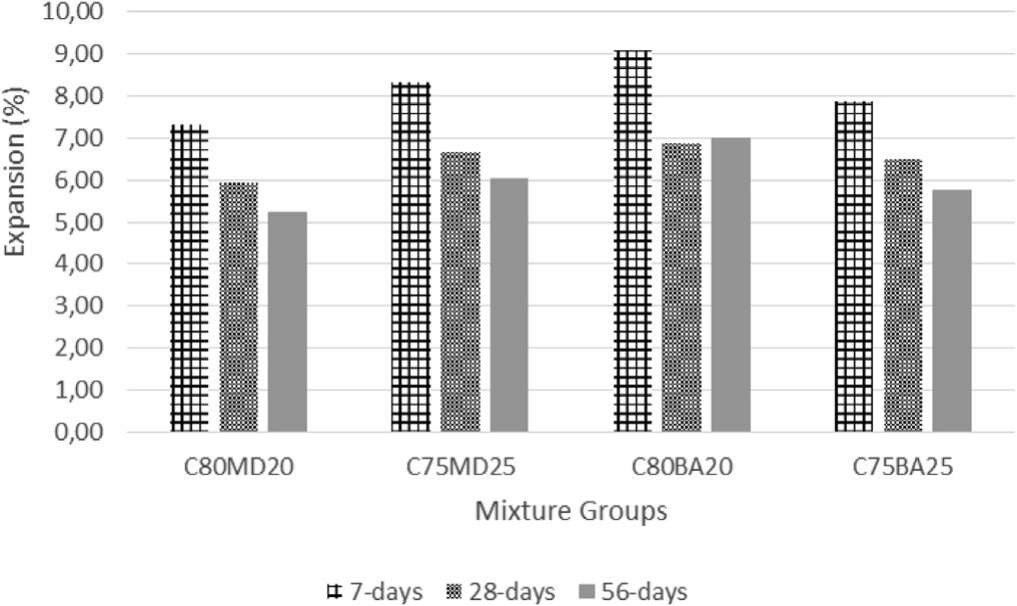
Expansion by sodium sulphate for bottom ash-marble-cement paste composites at 7-28-56 days of hardening.

**Fig. 9:**
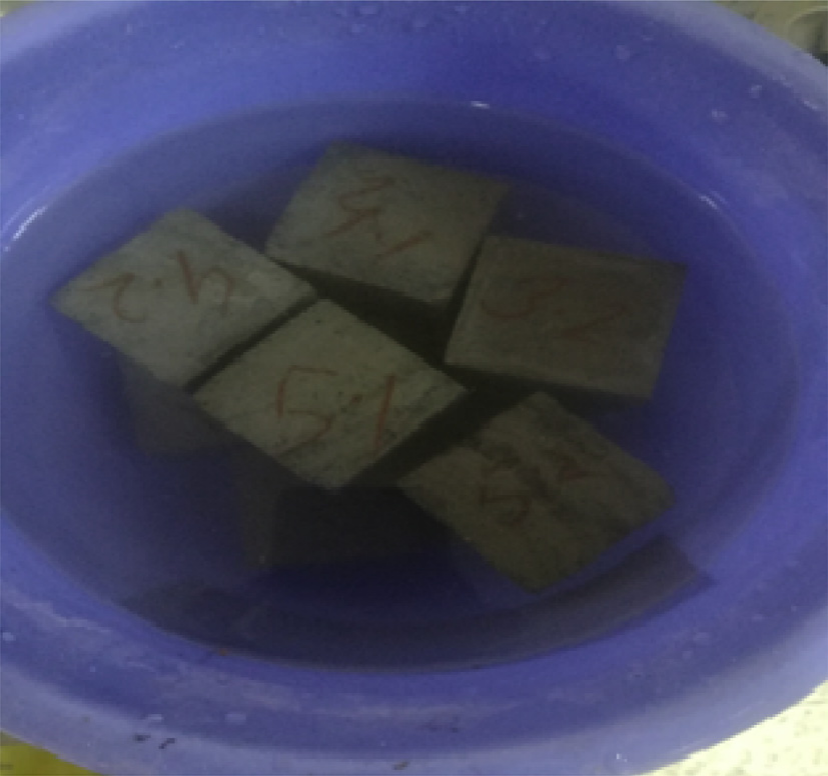
Selected samples immersed in a sulphate solution.

The data contains workability, strength, and durability properties of the marble-bottom ash-cement paste composites. Pure paste composites were prepared in a small laboratory, which produced samples. The samples were then assessed on the flow table test, as shown in Fig. 1a, for consistency. The laboratory produced samples are shown in Fig. 1b. The datasets compiled from the apparent specific gravity (ASG), porosity, bulk specific gravity (Dry), water absorption, unconfined compressive strength (UCS), flexural strength (FS) and sulphate resistance tests. The dataset is composed of physical, mechanical and durability test. Table 1 shows the fresh properties of the bottom ash-marble cement paste composites and Table 2 shows the measured data conducted during this research at 7, 28 and 90 days of hardening. Fig. 1a shows the flow table test for consistency (fresh state). Flow table and mini slump test data can help other researchers to identify the workability range of the tested samples. The 50-mm cubic and 40mmx40mmx160mm prisms of produced samples during this research are shown in Fig. 1b. In all Figures, MD denotes marble dust group, BA denotes bottom ash group and C denotes the cement. The numbers represent the mass percentages used in mixture proportioning. Fig. 2, shows bulk specific gravity (dry) values for all mixture groups. The Fig. 3, Fig. 4 represents porosity and water absorption values, which correspond to durability performance at 7, 28, and 56 days of hardening. Porosity and water absorption measurements are shown in Fig. 3, Fig. 4, and were chosen as durability parameters to evaluate the composites. Fig. 5 shows the apparent specific gravity values, which corresponds the pore characteristics of the composites. The compressive and flexural strength test values are shown in Fig. 6, Fig. 7. Fig. 8 shows the expansion by sodium sulphate for bottom ash-marble-cement paste composites at 7-28-56 days of hardening. Fig. 9 shows the selected samples immersed in a sulphate solution. Also, one excel worksheet is provided to help researchers to calculate the bulk specific gravity, porosity, water absorption and strength values and two video files are provided to show the first crack developing mechanisms for laboratory produced pure paste composites. More detailed information can be found both in supplementary Excel files and video files accompanying datasets and in Refs. [[1], [2], [3], [4]].

**Table 1:** Fresh Properties of bottom ash-marble cement paste composites.

Mix	Mini Slump (cm)	Flow table (cm)
C80MD20	3.3	20.5
C75MD25	4	22
C80BA20	4.2	30
C75BA25	4.3	31

**Table 2:** Measured data at 7, 28 and 56 days of hardening periods.

Age	Property	C80MD20	C75MD25	C80BA20	C75BA25
7	ASG	2.64	2.68	2.27	2.20
28		2.61	2.62	2.20	2.12
56		2.58	2.58	2.11	2.09
7	WA	19.80	24.30	27.40	30.20
28		16.20	20.30	22.90	24.50
56		15.80	18.80	20.20	22.10
7	BSG	1.88	1.76	1.53	1.44
28		1.83	1.71	1.46	1.39
56		1.80	1.69	1.42	1.37
7	Porosity	33.80	37.60	35.70	36.10
28		29.30	34.20	32.70	33.20
56		27.10	31.40	29.70	30.30
7	UCS	30.54	32.32	29.74	29.18
28		40.26	43.53	37.29	38.59
56		47.07	47.54	44.78	45.96
7	FS	4.13	4.26	4.04	3.96
28		5.87	6.08	5.47	5.40
56		6.57	6.65	6.25	6.42
7	Sulphate resistance expansion	7.32	8.33	9.09	7.87
28		5.94	6.67	6.88	6.49
56		5.25	6.03	7.01	5.77

Supplementary video related to this article can be found at https://doi.org/10.1016/j.dib.2020.105160.

The following is the supplementary data related to this article:

Video 13

## 2 Experimental design, materials, and methods

The data presented herein were obtained by incorporating a bottom ash and marble powder with cement. To satisfy the sustainability requirement, no treatment was applied to bottom ash and marble powder. The amount of moisture in marble powder were determined and the amount of water used in mixture proportioning were adjusted for every mixture groups. Marble powder was mainly composed of calcium carbonate and classified as limestone grade (calcium oxide: 44.3% and loss on ignition: 40.5%). The detailed information about chemical composition of marble can be found in Ref. [1]. The specific gravity of marble powder was 2.49 and its fineness was 3350 cm^2^/g. Bottom ash was obtained from local brick factory. The specific gravity was 1.44. Particles that passed through a 0.212 mm sieve was used. The ordinary Portland cement grade 42.5 was used. The specific gravity of cement was 3.15. The fineness of cement was 3650 cm^2^/g. The produced composites were designed such a way that no natural aggregates were utilized. The water to binder ratio for all groups was kept constant at 36.5%. This value was optimized based on previous studies [2,[5], [6], [7]]. Four different mixture groups were prepared. The mixture group C80MD20 comprised 80% cement and 20% marble dust. Similarly, C80BA20 comprised 80% cement and 20% bottom ash. Mixtures were cast in 50 mm cubic and 40 mm × 40 mm × 160 mm prismatic molds. The prepared samples were tested at 7, 28, and 56-days of hardening. Composites were evaluated based on the ACI report [8] and ASTM standards [9,10]. Twelve samples were cast for each test and curing age. The detailed mix proportions, experimental setup, and information can be found in Refs. [1,2], and datasets can be found in Refs. [3,4].
